# Sustainable Use of Pesticide Applications in Citrus: A Support Tool for Volume Rate Adjustment

**DOI:** 10.3390/ijerph14070715

**Published:** 2017-06-30

**Authors:** Cruz Garcerá, Alberto Fonte, Enrique Moltó, Patricia Chueca

**Affiliations:** Centro de Agroingeniería, Instituto Valenciano de Investigaciones Agrarias (IVIA), Carretera CV-315, Km 10.7, 46113 Moncada, Spain; crucecillah@gmail.com (C.G.); afontepolo@gmail.com (A.F.); molto_enr@gva.es (E.M.)

**Keywords:** dose rate, airblast sprayer, efficiency, efficacy, leaf area density, canopy volume

## Abstract

Rational application of pesticides by properly adjusting the amount of product to the actual needs and specific conditions for application is a key factor for sustainable plant protection. However, current plant protection product (PPP) labels registered for citrus in EU are usually expressed as concentration (%; rate/hl) and/or as the maximum dose of product per unit of ground surface, without taking into account those conditions. In this work, the fundamentals of a support tool, called CitrusVol, developed to recommend mix volume rates in PPP applications in citrus orchards using airblast sprayers, are presented. This tool takes into consideration crop characteristics (geometry, leaf area density), pests, and product and application efficiency, and it is based on scientific data obtained previously regarding the minimum deposit required to achieve maximum efficacy, efficiency of airblast sprayers in citrus orchards, and characterization of the crop. The use of this tool in several commercial orchards allowed a reduction of the volume rate and the PPPs used in comparison with the commonly used by farmers of between 11% and 74%, with an average of 31%, without affecting the efficacy. CitrusVol is freely available on a website and in an app for smartphones.

## 1. Introduction

The production and consumption of plant products play a very important role in the society, but the yield from plant production is continually threatened by harmful organisms, pests and diseases. It is essential to protect plants against such organisms in order to prevent their damage or a reduction in yield, and ensure both the quality of the products harvested as well as high agricultural productivity. Protecting plants from the effects of these organisms can be performed in many ways, but nowadays the most common methods are based on using plant protection products (PPPs). However, their use implies that PPP residues remain in food and find their way into the environment, consequently there is an important social pressure towards the development of measures for reducing the residues in food, minimizing the impact of pesticides on the environment and reducing and controlling the risks associated with their application.

One way to achieve these goals is through the rational application of PPPs by properly adjusting the amount of product to the actual needs and specific conditions of the application (vegetation to be treated, pest to be controlled, pesticide used and machinery). However, applying large quantities of product is fairly common today in order to ensure results, without taking into account that this practice normally entails an excessive release of products that remain in the food and pollute the environment. This practice also increases production costs.

In plant protection of tree orchards (pears, apples, citrus, olives…), vineyards, and high growing vegetables (hop, tomato...), which are known as 3-dimensional (3D) crops, contrary to the administration of pharmaceuticals to animals and/or humans in which the dose is expressed per kilo of body weight (mg/kg), current PPP labels registered in the southern regulatory zone (including Bulgaria, Greece, Spain, France, Italy, Cyprus, Malta, and Portugal) [1], are usually expressed as concentration (%; rate/hl) and/or as the maximum dose of product per unit of ground surface [2] with, in some cases, a registered maximum mix volume per unit of ground surface. However, although it is acknowledged that it is not appropriate to apply the same dosage of PPP in orchards with different target canopies. With respect to canopy size and/or leaf area density [3,4], i.e., young trees vs. 20-year-old trees, different cultivars, etc., there are no recommendations about how to adjust dose rates to account for these parameters. In fact, Garcerá et al. [5,6] demonstrated that there is a positive relationship between control efficacy and the application volume per unit volume of vegetation (L/m^3^), and at the same time, no significant relationship between the efficacy and the sprayed volume per unit ground area (L/ha) was found for either organophosphates or mineral oils against California red scale (*Aonidiella aurantii* Maskell (Hemiptera: Diaspididae)).

Different approaches for the adjustment of the application rate to the crop have been proposed. The first one was the “Tree Row Volume” (TRV) which determines rates based on the assumptions that each row of trees is a rectangular box whose volume could be used to calculate the volume space occupied by foliage per unit of ground surface (m^3^ of foliage per hectare), and that there is an optimum volume rate to reach the run-off point of the vegetation. The first proposal was made for apples by Byers et al. [7], considering an optimum volume rate of 0.094 L/m^3^ for a “standard apple orchard”, and that a “standard apple orchard” had 39,907 m^3^ of foliage/ha. Later on, it was found that when using TRV calibration, higher canopy densities reduced pesticide deposits. Therefore, adjustments for canopy density were advised when using TRV spray calibration guidelines [8,9,10,11,12]. In the case of orchards with row-end canopy profile different from a rectangular one (i.e., triangular), basic TRV calculations led to higher vegetation volume than the real one. TRV approach adjusted to the actual shape of the row-end canopy profile offered better results [13,14,15,16]. Furness et al. [17] proposed a simpler method called “Unit Canopy Row” (UCR), based on the definition of the minimum volume of application required to drip 100 m^3^ of vegetation (1 m high × 1 m wide × 100 m long), expressed in L/100 m of row length. The UCR method achieved good results both in vineyards and citrus orchards [18,19].

Simpler methods, considering different parameters to characterize just the vertical surface of the crop row have also been developed. Grout [20] elaborated a table of spray volumes per meter of row and meter of tree height for a range of applications to citrus trees. Another method based on “Canopy Height” (CH) was introduced using solely this parameter as the dominant crop parameter, making the assumption that uniform row distance doses can be adjusted to the most important crop-specific parameters [21,22]. According to Pergher and Petris [23], this model would hold only if the remaining parameters (leaf area density, canopy width, row spacing) are constant, which might be true in very few cases. According to Hucorne [24], Belgium made the first attempts to use the “Leaf Wall Area” (LWA, m^2^ treated area/ha) as the dose expression of PPPs for fruit production in 1996. It considers the vegetation as a vertical wall facing the spray. Koch [25] stated that when spraying orchards, nozzles are directed to the tree canopies, so the canopy height defines the “treated or oversprayed area”, defined as the vertical plane, parallel to the tree row, that the delivered spray fluid has to pass through before droplets reach the target [26]. This parameter is proposed to be the basis for dose expression of PPPs at the European level [26,27,28,29]. However, TRV and LWA do not take into account real tree row-end profile, leaf density, etc., therefore their use could lead to an inaccurate dosage [17,23,30,31,32].

In the last years, PPP dose adjustment tools for 3D crops have been developed for orchards (SARDI/PIRSA [33], Pesticide Adjustments to Crop Environment (PACE) [34,35], and Dosafrut [32]), for vineyards (SARDI/PIRSA, and Dosaviña [36]), for citrus (SARDI/PIRSA, and Dosacitric [37]), and for greenhouse tomato crops (GreenRate [38]). These tools take into account parameters of vegetation (basic TRV, TRV adjusted to the row profile, estimated Leaf Area Index (LAI), etc.) and others factors related to the efficiency of the application and the minimum deposit to be achieved on the leaves. Regarding the latter parameter, in the case of the SARDI/PIRSA tool, the recommendation is based in the water volume per UCR established to achieve the run-off point in the different crops considered; in the case of the PACE tool, the system combines a generalized dosage model that minimize the variation of tree average deposits and a dataset of target structures of regional exemplars where efficient and efficacious use of pesticide was obtained at the label-recommended dose rate; in other cases (“Dosaviña”, “Dosafrut” and “Dosacitric” tools), the optimum number of droplets per unit of leaf area and optimum droplet size are set; and in the case of the “GreenRate” tool, a desired deposit is established based on the average foliar deposition obtained in previous studies in greenhouse tomato crops, taking for granted that the application with spray guns established by the operator offers good biological efficacy.

The objective of the present work was to design a tool to help citrus growers in the process of choosing the appropriate volume rate to be applied in their orchards, taking into account the size, the geometry and the foliar density of the target canopies, the pest to be controlled, the product to be applied, and the efficiency of airblast applications in citrus. The fundamentals of the tool named CitrusVol and its evaluation are presented in this manuscript.

## 2. CitrusVol

### 2.1. Fundamentals

The tool is based on achieving a minimum deposit on the target surface (leaves, wood, fruit) for achieving maximum efficacy of control according to the pest/disease and the way of action of the applied plant protection product (PPP). This minimum deposit was calculated from the models determined in the laboratory that relate the amount of product deposited, obtained varying the mix volume at the label concentration, how it is deposited and how it affects the control of the pest, setting the differences between developmental stages [39,40]. These models were developed for California red scale, *Aonidiella aurantii* Maskell (CRS), which was selected as a reference pest because it is a key pest in worldwide citrus production [41] and has characteristics that makes it difficult to be controlled (shielded body, location preferences, low mobility, tendency to aggregate in colonies, survival in wood between seasons, etc.). These models were subsequently validated in field conditions [5,6], and they showed that to achieve the maximum efficacy (90%) in treatments against first CRS generation, in which young stages are predominant [41], a deposition level (***D***, µL/cm^2^) of 1.01 µL/cm^2^ was necessary when applying organophosphates, which were selected as the reference for contact PPP, and this level increased to 3.41–4.72 µL/cm^2^ when applying mineral oils, the reference for suffocating PPP. For applications against next generations, in which there is heterogeneity of stages, ***D*** was between 3.41 and 4.72 µL/cm^2^ for organophosphates and it was of 4.72 µL/cm^2^ for oils. From these values, to be on the side of safety, a deposit of 3.41 µL/cm^2^ was considered when applying organophosphates, and of 4.72 µL/cm^2^ when applying mineral oils. Besides, it is acknowledged that the survival of pests in field conditions is lower than in laboratory conditions, due to the more adverse climatic conditions, natural enemies, etc. [42,43,44]. For this reason, 80% of the laboratory deposits were considered in the tool. Based on these results, when the user of the tool selects the product, the minimum deposit to be used in the subsequent calculations is selected. This database will be updated as new models of PPP efficacy-deposition will be developed.

Once the minimum deposits are set, the theoretical volume rate to be used in the planned application (***V***, L/ha) is calculated with Equation (1).

***V*** = ***D*** × ***S_W_*** × ***N*** × ***f_lab-field_*** (1)

where ***D*** (µL/cm^2^) is the minimum deposition level, ***S_W_*** (m^2^ leaf /tree) is the total leaf surface per tree to be wet, ***N*** is the number of trees in a hectare, which is calculated with the Equation (2), taking into account tree and row spacing, and ***f_lab-field_*** (–) is the factor introduced to account for the differences between laboratory and field survival.

***N*** = 10000/(***sp_tree_*** × ***sp_row_***) (2)

where ***sp_tree_*** (m) is the distance between tree trunks in a row (spacing within row), and ***sp_row_*** (m) is the distance between rows (row spacing).

The total leaf surface per tree to be wet (***S_W_***, m^2^ leaf /tree) was calculated with Equation (3) from the total one-side leaf surface per tree (***S***, m^2^ leaf one-side/tree), taking into account that the two sides of the leaves had to receive the sprayed product, for which ***S*** is multiplied by 2, and that the different targets of PPP applications (pest and/or disease) have different requirements regarding which part of the canopy has to be wet, for which the target factor ***f_target_*** (–) is included.

***S_W_*** = 2 × ***S*** × ***f_target_*** (3)

where ***S*** (m^2^ leaf one-side/tree) is the total leaf one-side surface per tree and ***f_target_*** (–) is the target factor.

To define ***f_target_***, PPP applications were differentiated depending on the percentage of the canopy that has to be covered, differentiating between “internal”, “intermediate” and “external” applications. Internal applications have to reach the entire canopy, intermediate applications have to reach two-thirds of the canopy, and external applications have to reach one-third of the canopy. Based on these requirements, and taking into account the ellipsoidal shape of the citrus canopy, the corresponding values of ***f_target_*** for each group were calculated (Table 1). The different pests/diseases were allocated to the corresponding application type depending on the covering requirements. This allocation was decided with the advice of the plant protection research group of the Instituto Valenciano de Investigaciones Agrarias (IVIA).

The user selects the specific target (pest/disease) of the planned application and the tool includes in the calculations the corresponding value of ***f_target_***.

With respect to the total leaf surface per tree ***S*** (m^2^ leaf/tree), this was calculated with the Equation (4).

***S*** = ***LAD*** × ***VT*** (4)

where ***VT*** (m^3^/tree) is the average apparent volume of trees considering that the canopy of citrus has an ellipsoidal shape, and ***LAD*** (m^2^ leaf/m^3^ canopy) is the leaf area density.

With respect to the procedure for quantification of ***LAD***, it was considered that it included both the effect of the pruning level of the orchard and the cultivar, and the user of the tool has to choose between the options offered. Concerning pruning, three levels were considered: ‘severe’, ‘normal’ and ‘without pruning’. Concerning the cultivar, they were grouped based on their mean density. Three groups were considered: ‘low density cultivars’, ‘medium density cultivars’ and ‘high density cultivars’. Some examples from each group are given in the tool to facilitate the choice to the users. The inclusion of the different cultivars in the corresponding group was decided with the advice of the citrus research group of the IVIA. Depending on the choices of the user of the tool on these two parameters, a value of ***LAD*** from the included database (Table 2) is selected and included in the calculations.

**Figure 1:**
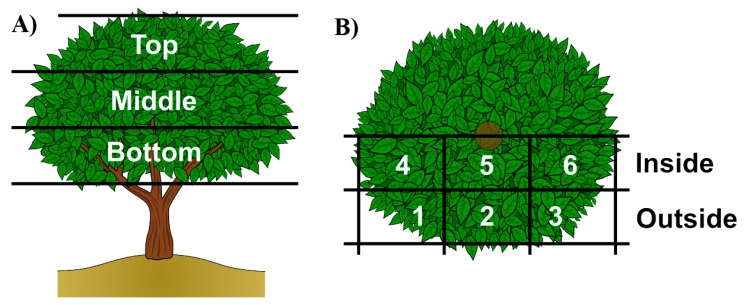
(**A**) Side view of a standard tree. Distribution of assessment zones in height; (**B**) Top view of a standard tree. Distribution of assessment zones at each height.

To define the reference values of ***LAD*** to be included in the tool, ***LAD*** was assessed in different orchards with different level of pruning. The assessment was made by sampling leaves in different quadrants of the canopy, resulting from dividing the canopy as shown in Figure 1.

In each quadrant, a cube of 70 × 70 × 70 cm (0.343 m^3^) was installed, taking care not to vary the initial vegetative structure, and all the leaves inside the delimited volume were collected and weighed to obtain the ratio ‘g of leaves/m^3^ of canopy’. Afterwards, in the laboratory, a sample of 10 leaves of each quadrant was weighed with an analytical balance (XR 205 SM-DR, Precisa Instruments Ltd., Dietikon, Switzerland) and their leaf area was determined. To do this, they were digitized by scanning and the resulting image was analyzed by image analysis (Matrox Inspector, v. 2.2, Matrox Electronic Systems Ltd., Dorval, QC, Canada). With these values, the ratio ‘cm^2^ of leaves/g’ of the leaves of each quadrant was calculated. Using the ratios m^2^/m^3^ and cm^2^/g obtained for each quadrant, the leaf surface per unit volume of vegetation (m^2^ leaf/m^3^ vegetation) of each of them was calculated. Three repetitions were performed in each orchard. Mean value of each orchard was calculated afterwards and included in the database of the tool.

For the calculation of the apparent volume of trees, ***VT***, the tool includes two cases. If the average diameter of the trees along the row is equal or larger than the distance between trees in a row, ***VT_1_*** is calculated with Equation (5). If the average diameter of the trees along the row is shorter than the distance between trees in a row, ***VT_2_*** is also calculated with Equation (6). This case was considered to take into account the orchards with gaps between trees that are sprayed with equipment without technology to detect the vegetation, which is fairly common.

***VT_1_*** = 1/6 π × ***h*** × ***Ø_across the row_*** × ***Ø_along the row_*** (5)

***VT_2_*** = 1/6 π × ***h*** × ***Ø_across the row_*** × ***sp_tree_*** (6)

where ***h*** (m) is the average canopy height (considering the height from the bottom of the canopy (not the ground) to the top of the canopy), ***Ø_across the row_*** (m) is the average diameter of the trees measured perpendicularly to the row, ***Ø_along the row_*** (m) is the average diameter of the trees measured in parallel to the row, and ***sp_tree_*** (m) is the distance between tree trunks in a row.

Up to this point, the theoretical volume rate to be used in the planned application is calculated. In order to give the recommended volume rate (***V_R_***, L/ha), the efficiency of the application is also taken into account and calculated through Equation (7).

***V_R_*** = ***V***/***f_E_*** (7)

where ***f_E_*** (–) is the efficiency factor, which makes reference to the part of spray that is delivered by the airblast sprayer and reaches the intended target canopy. According to previous studies, in pesticide applications performed in Mediterranean citrus orchards with conventional air-blast sprayers, almost 50% of the spray reaches the intended canopy [45]. In that work, the spray mixture was water + tracer, if adding an adjuvant, which is a common practice among farmers and is a usual ingredient of commercial pesticides, the efficiency is considered to increase in 20% respect to when applying water alone [46,47]. Therefore, a ***f_E_*** = 0.6 is assumed in the tool.

With all these values, the recommended volume rate is calculated. There are two possible cases, as mentioned before: 

If ***Ø_along the row_*** ≥ ***sp_tree_***, ***VT_1_*** is calculated with Equation (5), and this value is used to calculate ***S_1_***, ***S_W1_***, ***V_1_*** and ***V_R1_*** with the corresponding equations. In this case, ***V_R1_*** would be the recommended volume rate.

If ***Ø_along the row_*** < ***sp_tree_***, on the one hand, ***VT_1_***, ***S_1_***, ***S_W1_***, ***V_1_*** and ***V_R1_*** are calculated with the corresponding equations, and on the other hand, ***VT_2_*** is calculated with Equation (6), and ***S_2_***, ***S_W2_*** and ***V_R2_*** are subsequently calculated with the corresponding equations based on this value. In this case, the tool indicates that ***V_R1_*** would be the recommended volume rate in case of using a sprayer with technology to detect the vegetation, that is to say, that the nozzles are only operative in front of the canopy [48], and ***V_R2_*** would be the recommended volume rate in case of using a sprayer without this technology. Besides, the tool indicates the percentage of saved amount of volume that would be obtained in case of using sprayers with technology to detect the vegetation.

As stated, these recommendations are based on the achievement of a minimum deposit on the target surface, but they do not take into account the limitations of volume rate or dose that appear in labels of some commercial products, which are usually based on limitations set in some parts of the registration dossier which are not related with the biological control. Therefore, besides the recommended volume rate, the following warning message appears “Check the technical data sheet of the product to verify if it is authorized for this use and/or if there are limits of maximum application volume rate or maximum dose”.

### 2.2. User Interface

The tool is freely available and it is included in the citrus Integrated Pest Management (IPM) website [49] and in the citrus IPM app Gipcitricos IVIA developed for smartphones [50]. The input data to be included by the user are:

- Canopy characteristics:
  - ***h*** (m) is the average canopy height
  - ***Ø_across the row_*** (m) is the average diameter of the trees measured perpendicularly to the row
  - ***Ø_along the row_*** (m) is the average diameter of the trees measured in parallel to the row
  - Cultivar, selection from a drop-down menu based on Table 2
  - Pruning level, selection from a drop-down menu based on Table 2
- Orchard characteristics (framework)
  - ***sp_tree_*** (m) is the distance between tree trunks in a row (tree spacing)
  - ***sp_row_*** (m) is the distance between tree trunks across a row (row spacing)
- Application specifications
  - Target, selection from a drop-down menu based on Table 1
  - Product, selection from a drop-down menu with all the registered products for citrus

**Figure 2:**
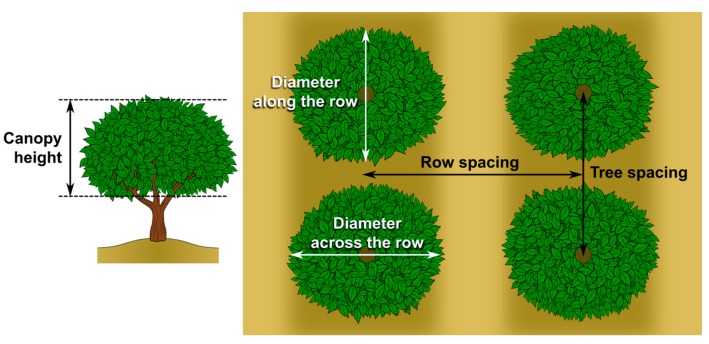
Parameters of the framework and the canopy size of the target orchard that growers have to measure and include in the tool.

A diagram showing the parameters of the framework and the canopy size that growers have to measure is included in the tool (Figure 2).

**Figure 3:**
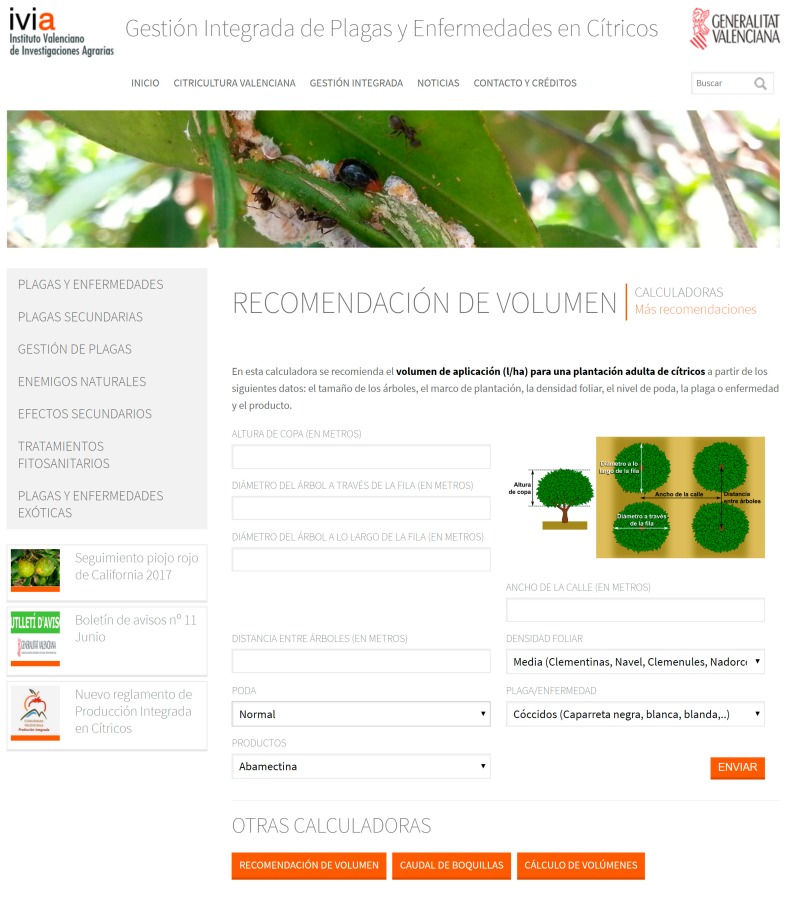
Interface of CitrusVol in the website [49].

The tool is presented in a friendly interface (Figure 3). It is written in Spanish and it is going to be translated to English and to languages of other citrus areas (Italian, Portuguese, Greek).

## 3. Field Evaluation of the Tool to Determine the Volume Rate for Spraying Citrus

The CitrusVol tool was evaluated through field tests carried out during the season 2015–2016 in seven commercial Clementine orchards located in Valencia (Spain). The characteristics of each orchard are shown in Table 3. Field tests consisted of comparing conventional rates (conventional treatment, Vc) used by farmers with the volume rates recommended by the CitrusVol tool (adjusted treatment, Va). This comparison was made for applications against *Aonidiella aurantii* (California red scale, CRS) and *Tetranychus urticae* (two-spotted spider mite, TSM). Treatments were applied by means of conventional airblast sprayers from the farm. A total of 14 PPP applications were carried out, with the sprayer set up shown in Table 4. In each orchard, the number of open nozzles for the Vc applications was selected by the farmers. They also selected the combination of nozzle sizes in the nozzle manifold to give the expected volume rate, following the conventional regulation of the sprayer in each case. For the Va applications, the number of open nozzles in each orchard was selected by a visual assessment of the spray cloud to fit it to the canopy size and shape. Afterwards, the combination of nozzle sizes of the same model used for the Vc applications in each orchard was selected to give the adjusted volume rate. Nozzle sizes were not the same for the whole manifold in any case.

The percentages of reduction of mix volume between conventional treatments and adjusted treatments were calculated (Table 5). Percentages of reduction were between 11.61% and 74.08% with an average reduction of 31.5%. Because of the fact that the PPP labels for citrus in Spain are usually expressed as concentration, the percentage of reduction of PPP are the same that the percentage of reduction of mix volume. The quantity of PPP savings is shown in Table 5.

The reduction of the use of mix volume per hectare also implies a reduction on the number of tank refills, and therefore a reduction of operational time. The time savings in each application are shown in Table 6. Time savings increase when the area to be spray increases due to economies the scale.

In terms of efficacy, quality of fruit at harvest in each orchard was assessed by measuring the percentage of cull fruit due to each pest with each treatment. Cull threshold due to two-spotted spider mite was considered as the fruit that presented extensive skin damage, at least in the apex and stem zones. Cull threshold due to California red scale was considered as the fruit that presented three or more scales. In all the orchards, the values of cull fruit were very low and similar between treatments (Table 7).

## 4. Conclusions

The CitrusVol tool was designed to help citrus growers in the process of choosing the appropriate volume rate to be applied in their orchards, taking into account the size, the geometry and the foliar density of the target canopies, the pest/disease to be controlled and the product to be applied. It is based on the relationships between the quantity of deposited PPP, how it is deposited on the citrus leaves, and how it affects the control of the pest and/or the disease, therefore it allows the rational adjustment of the amount of mix to be applied in PPP applications in citrus crops.

The determination of the optimal application volume rate with the tool allows the application efficiency to be increased without affecting the control efficacy, whilst minimizing losses and environmental exposure. It also provides a reduced water footprint, decreases PPP costs and operational time, and reduces residues on fruit, with the subsequent reduction of PPP in the agri-food chain.

## Figures and Tables

**Table 1:** Targets allocated with each type of application and the corresponding calculated value of ***f_target_*** (–).

Application	Target		*f_target_*
Internal	Pests	- Armored scales (California red scale *Aonidiella aurantii*, oleander scale *Aspidiotus nerii*…) - Mites (two-spotted spider mite *Tetranychus urticae*, red mite *Panonychus citri*…) - Mealybugs (citrus mealybug *Planococcus citri*, citrophilus mealybug *Pseudococcus citri*…) - Soft scales (black scale *Saissetia oleae*, Chinese wax scale *Ceroplastes sinensis*, brown soft scale *Coccus hesperidum*…) - Cottony cushion scale (*Icerya purchasi*) *	1
	Diseases	- Citrus brown spot (*Alternaria alternata*) (applications performed in autumn)	
Intermediate	Pests	- Thrips (*Pezothrips kellyanus*) - Aphids (cotton aphid *Aphis gossypii*, green citrus aphid *Aphis spiraecola*…) - Woolly whitefly (*Aleurothrixus floccosus*) - Moths (citrus leafminer *Phyllocnistis citrella*, carob moth *Ectomyelois ceratoniae*, *Cryptoblabes guinidiella*…)	0.75
	Diseases	- Citrus brown spot (*Alternaria alternata*) (applications performed in spring) - Brown rot of citrus fruit (*Phytophthora spp.*) - Foot rot and gummosis (*Phytophthora spp.*) **	
External	Pest	- Mediterranean fruit fly (*Ceratitis capitata*) ***	0.49

* At the time of writing this manuscript, there were no products authorized against this pest in Spain, so when it is selected the following warning message appears “There are no authorized products”. In case of future authorization of any product, this warning message would not appear and ***f_target_*** = 1 would be used; ** The applications against this disease should be carried out directly on the trunk and main branches in advance of the onset of infections, so when it is selected the following warning message appears “Applications directly on the trunk”; *** At the time of writing this manuscript, some products were authorized only as bait treatment, so when these products are selected for controlling this pest the following message appears “Application as bait treatment”.

**Table 2:** Cultivars allocated within each group and the estimated mean values of leaf area density (***LAD***; m^2^ leaf/m^3^ canopy) for each combination of pruning level and cultivar.

Cultivar	Examples Included in the Tool	Pruning Level		
		Severe	Normal	Without Pruning
Low density	Satsuma group (Owari, Okitsu) Lemon spp. (lemon cv. ‘Fino’)	2.5	2.9	3.3
Medium density	Clementine group (Clemenules, Marisol, Oronules, Nadorcott), Hybrid group (Nova, Orri) Navel group (Washington, Lane late)	3.3	3.7	4.1
High density	Hybrid group (Fortune, Garbí, Moncada)	4.1	4.6	5

**Table 3:** Characteristics of trial orchards.

Orchard	Cultivar	Location Geografic Coordinates	Tree Spacing (m) (sp_row_ × sp_tree_)	Canopy Dimensions (m) (Height × Ø_across row_ × Ø_along row_)	Apparent Canopy Volume (*VT_1_*, m^3^/tree)
P1	Clemenules	39° 26′ 32.3″ N, 0° 33′ 23.1″ W	6 × 3	2.51 × 4.33 × 3.08	17.53
P2	Oronules	39° 26′ 42.9″ N, 0° 32′ 17.9″ W	7 × 2	2.12 × 4.26 × 2.30	10.88
P3	Clemenules	39° 39′ 11.1″ N, 0° 18′ 25.1″ W	6.8 × 5	2.15 × 3.39 × 3.34	12.75
P4	Clemenules	38° 56′ 46.3″ N, 0° 14′ 15.1″ W	5.5 × 5	2.38 × 4.28 × 4.57	24.37
P5	Clemenules	38° 56′ 55.9″ N, 0° 14′ 31.7″ W	6 × 2	1.99 × 2.93 × 1.96	5.98
P6	Clemenules	39° 43′ 42.6″ N, 0° 35′ 27.7″ W	6.5 × 2.5	2.47 × 3.90 × 2.60	13.11
P7	Clemenules	39° 43′ 57.8″ N, 0° 35′ 32.5″ W	6.5 × 3.5	2.45 × 4.84 × 3.70	22.97

***sp_tree_*** (m): distance between tree trunks in a row (spacing within row); ***sp_row_*** (m): distance between rows (row spacing) ), ***Ø_across row_*** (m): average diameter of the trees measured perpendicularly to the row; ***Ø_along row_*** (m): average diameter of the trees measured in parallel to the row; ***VT_1_*** (m^3^/tree): Apparent canopy volume calculated with Equation (5).

**Table 4:** Plant protection product (PPP) applications carried out during and set up of the sprayers used.

Orchard	Application Date	Pest	Pressure (Bar)	Forward Speed (km/h)	Air Volume (m^3^/h)	Number of Open Nozzles	
						Vc	Va
P1	27 May 2016	CRS (first generation)	8	1.32	55342.15	38	30
	27 July 2016	CRS (second generation) and TSM	8	1.32	55342.15	38	30
	11 October 2016	TSM	8	1.32	55342.15	38	30
P2	26 May 2016	CRS (first generation)	8	1.32	55342.15	38	28
	22 June 2016	TSM	8	1.73	55342.15	38	30
	09 August 2016	CRS (second generation) and TSM	8	1.32	55342.15	38	28
	09 September 2016	TSM	8	1.73	55342.15	38	30
P3	09 June 2016	CRS (first generation)	13	1.75	101248.29	26	18
P4	31 May 2016	CRS (first generation)	9	1.92	54828.19	36	14
P5	31 May 2016	CRS (first generation)	9	1.92	54828.19	34	18
P6	16 June 2016	CRS (first generation)	8	1.53	89268.43	26	22
	12 August 2016	CRS (second generation)	8	1.48	89268.43	26	22
P7	14 June 2016	CRS (first generation)	8	1.53	89268.43	26	26
	11 August 2016	CRS (second generation)	8	1.48	89268.43	26	26

CRS: California red scale; TSM: Two-spotted spider mite; Vc: conventional treatment; Va: Adjusted treatment.

**Table 5:** Water volume used in the applications and the percentage of mix volume reduction and the PPP savings due to the use of the CitrusVol tool.

Orchard	Application Date	Pest	Active Ingredient of PPP	PPP Concentration (%)	Water Volume			PPP Savings (kg PPP/ha or L PPP/ha)
					Vc (L/ha)	Va (L/ha)	Reduction (%)	
P1	27 May 2016	**CRS** (first generation)	Chlorpyrifos	0.20	4905	3255	33.64	3.30
			Pyriproxyfen	0.05				0.83
			Abamectin	0.04				`0.66
			Spirodiclofen	0.02				0.33
	27 July 2016	**CRS** (second generation) and **TSM**	Abamectin	0.10	4905	3255	33.64	1.65
			Spirotetramat	0.04				0.66
			Spirodiclofen	0.02				0.33
	11 October 2016	**TSM**	Abamectin	0.10	4905	3255	33.64	1.65
P2	26 May 2016	**CRS** (first generation)	Chlorpyrifos	0.20	4204	2800	33.39	2.81
			Etoxazole	0.05				0.70
			Pyriproxyfen	0.05				0.70
			Abamectin	0.04				0.56
	22 June 2016	**TSM**	Abamectin	0.10	3215	2476	22.98	0.74
			Spirodiclofen	0.02				0.15
	09 August 2016	**CRS** (second generation) and **TSM**	Abamectin	0.10	4204	2800	33.39	1.40
			Spirotetramat	0.04				0.56
			Tetrazine	0.02				0.28
	09 September 2016	**TSM**	Abamectin	0.10	3215	2476	22.28	0.74
			Spirodiclofen	0.02				0.15
P3	09 June 2016	**CRS** (first generation)	Abamectin	0.04	3264	2294	29.70	0.39
			Spirotetramat	0.02				0.19
			Clofentezine	0.01				0.10
P4	31 May 2016	**CRS** (first generation)	Spirotetramat	0.04	7311	3011	58.82	1.72
P5	31 May 2016	**CRS** (first generation)	Chlorpyrifos	0.20	6702	1737	74.08	9.93
			Pyriproxyfen	0.08				3.97
			Spirodiclofen	0.02				0.99
P6	13 June 2016	**CRS** (first generation)	Chlorpyrifos	0.27	3200	2535	20.81	1.80
			Abamectin	0.13				0.86
			Pyriproxyfen	0.07				0.47
			Clofentezine	0.02				0.13
	12 August 2016	**CRS** (second generation)	Spirotetramat	0.04	3318	1628	20.81	0.68
P7	14 June 2016	**CRS** (first generation)	Chlorpyrifos	0.27	3468	3065	11.61	1.09
			Abamectin	0.13				0.52
			Pyriproxyfen	0.07				0.28
			Clofentezine	0.02				0.08
	11 August 2016	**CRS** (second generation)	Spirotetramat	0.04	3595	3177	11.61	0.17

CRS: California red scale; TSM: Two-spotted spider mite; PPP: plant protection product; Vc: conventional treatment; Va: Adjusted treatment.

**Table 6:** Time savings of tank refill for each application and for different size areas to be sprayed, considering an average refilling time of 40 min per tank, including equipment transit time to and from the water source [51].

Orchard	Application Date	Tank Capacity (L)	Number of Tanks/ha		Time Savings of Tank Refill (h/ha)	Time Savings of Tank Refill (h/10 ha)	Time Savings of Tank Refill (h/100 ha)
			Vc	Va			
P1	27 May 2016	1500	4	3	0.67	7.33	73.33
	27 July 2016		4	3	0.67	7.33	73.33
	11 October 2016		4	3	0.67	7.33	73.33
P2	26 May 2016	1500	3	2	0.67	6.67	62.66
	22 June 2016		3	2	0.67	3.33	32.66
	09 August 2016		3	2	0.67	6.67	62.66
	09 September 2016		3	2	0.67	3.33	32.66
P3	09 June 2016	2000	2	2	0	3.33	32.66
P4	31 May 2016	2000	4	2	1.33	14	143.33
P5	31 May 2016	2000	4	1	2	16.67	166
P6	16 June 2016	3000	2	1	0.67	1.33	14.66
	12 August 2016		2	1	0.67	4	37.33
P7	14 June 2016	3000	2	2	0	0.67	8.66
	11 August 2016		2	2	0	0.67	9.33

Vc: conventional treatment; Va: Adjusted treatment.

**Table 7:** Percentage of cull fruit (%) (mean (standard error)) at harvest in each orchard due to each pest with each treatment.

Orchard	Two-Spotted Spider Mite		California Red Scale	
	Vc	Va	Vc	Va
P1	0	0.25 (0.25)	0	0
P2	1.00 (0.58)	0.75 (0.41)	0.25 (0.25)	1.00 (0.58)
P3	-	-	0	0.25 (0.25)
P4	-	-	3.50 (1.15)	3.75 (1.30)
P5	-	-	0.25 (0.25)	0
P6	-	-	0	0
P7	-	-	0	0

Vc: conventional treatment; Va: Adjusted treatment.
